# Population-Based Incidence Rates of Diarrheal Disease Associated with Norovirus, Sapovirus, and Astrovirus in Kenya

**DOI:** 10.1371/journal.pone.0145943

**Published:** 2016-04-26

**Authors:** Kayoko Shioda, Leonard Cosmas, Allan Audi, Nicole Gregoricus, Jan Vinjé, Umesh D. Parashar, Joel M. Montgomery, Daniel R. Feikin, Robert F. Breiman, Aron J. Hall

**Affiliations:** 1 Division of Viral Diseases, National Center for Immunization and Respiratory Diseases, Centers for Disease Control and Prevention, Atlanta, Georgia, United States of America; 2 Oak Ridge Institute for Science and Technology, Oak Ridge, Tennessee, United States of America; 3 Division of Global Health Protection, Center for Global Health, Centers for Disease Control and Prevention, Nairobi, Kenya; 4 Kenya Medical Research Institute, Center for Global Health Research, Nairobi, Kenya; Aga Khan University Hospital Nairobi, KENYA

## Abstract

**Background:**

Diarrheal diseases remain a major cause of mortality in Africa and worldwide. While the burden of rotavirus is well described, population-based rates of disease caused by norovirus, sapovirus, and astrovirus are lacking, particularly in developing countries.

**Methods:**

Data on diarrhea cases were collected through a population-based surveillance platform including healthcare encounters and household visits in Kenya. We analyzed data from June 2007 to October 2008 in Lwak, a rural site in western Kenya, and from October 2006 to February 2009 in Kibera, an urban slum. Stool specimens from diarrhea cases of all ages who visited study clinics were tested for norovirus, sapovirus, and astrovirus by RT-PCR.

**Results:**

Of 334 stool specimens from Lwak and 524 from Kibera, 85 (25%) and 159 (30%) were positive for norovirus, 13 (4%) and 31 (6%) for sapovirus, and 28 (8%) and 18 (3%) for astrovirus, respectively. Among norovirus-positive specimens, genogroup II predominated in both sites, detected in 74 (87%) in Lwak and 140 (88%) in Kibera. The adjusted community incidence per 100,000 person-years was the highest for norovirus (Lwak: 9,635; Kibera: 4,116), followed by astrovirus (Lwak: 3,051; Kibera: 440) and sapovirus (Lwak: 1,445; Kibera: 879). For all viruses, the adjusted incidence was higher among children aged <5 years (norovirus: 22,225 in Lwak and 17,511 in Kibera; sapovirus: 5,556 in Lwak and 4,378 in Kibera; astrovirus: 11,113 in Lwak and 2,814 in Kibera) compared to cases aged ≥5 years.

**Conclusion:**

Although limited by a lack of controls, this is the first study to estimate the outpatient and community incidence rates of norovirus, sapovirus, and astrovirus across the age spectrum in Kenya, suggesting a substantial disease burden imposed by these viruses. By applying adjusted rates, we estimate approximately 2.8–3.3 million, 0.45–0.54 million, and 0.77–0.95 million people become ill with norovirus, sapovirus, and astrovirus, respectively, every year in Kenya.

## Introduction

Although global deaths from diarrheal disease have decreased from 2.6 million to 1.3 million between 1990 and 2013 [1], it remains a major health concern, particularly in Africa. Diarrheal illness was estimated to be one of the top causes of years of life lost in sub-Saharan Africa in 2013, along with HIV/AIDS, lower respiratory infections, and malaria [1]. Young children are known to be the most affected population, and there were an estimated 450 million diarrheal episodes among children <5 years of age in 2010 in Africa [2]. The burden of rotavirus, which remains the leading cause of severe diarrheal disease in children worldwide, has been well characterized in both developed and developing countries [3–8]. Although norovirus, sapovirus, and astrovirus are also recognized as contributors to diarrheal disease [9], population-based rates of disease associated with these viruses are not available in sub-Saharan African countries. With the expected decline of rotavirus disease after vaccine implementation [10–13], monitoring for other diarrheal pathogens will be important to assess changes in their relative burden in order to implement appropriate public health measures, especially in regions with high incidence of diarrheal diseases.

To help fill this gap, we aimed to estimate population-based rates of diarrheal illness associated with norovirus, sapovirus, and astrovirus in Kenya. We used a unique active surveillance system for infectious disease syndromes implemented by the Kenya Medical Research Institute (KEMRI) and the United States Centers for Disease Control and Prevention (CDC) to achieve this goal.

## Materials and Methods

### Population-based infectious disease surveillance

**Fig 1:**
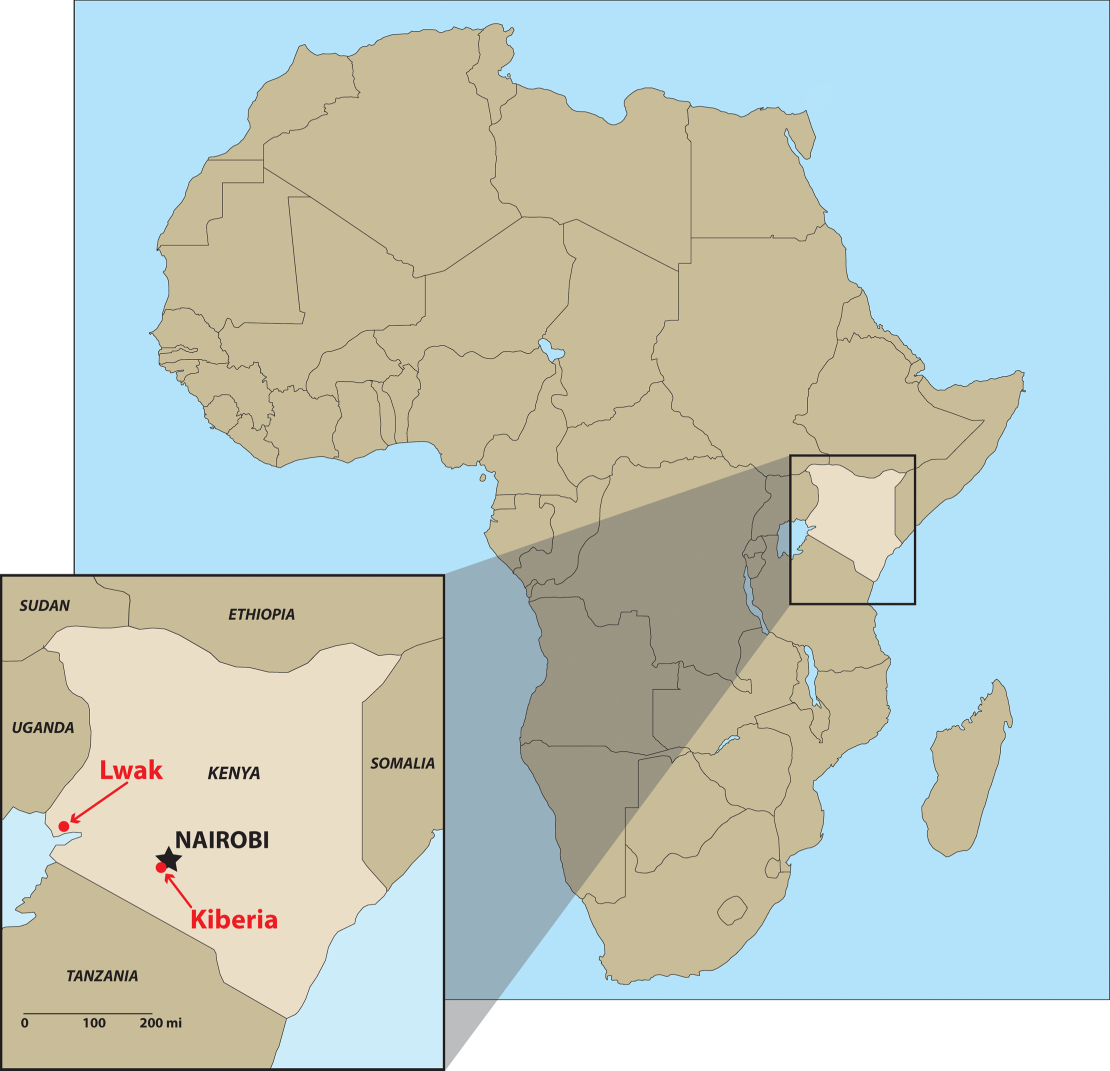
Map of the study sites in Kenya.

The population-based infectious disease surveillance (PBIDS) system was launched in late 2005 by CDC and KEMRI. The system encompassed two sites in Kenya: Lwak and Kibera (Fig 1). Lwak is a rural location in Nyanza province (current Kisumu Country) near Lake Victoria in Western Kenya, which is one of the poorest areas in the country. Kibera is an urban slum in Nairobi, which is the capital and the largest city in Kenya. The geographic characteristics of these surveillance areas have been described previously [14, 15]. The study periods for this analysis was from June 2007 to October 2008 (one year and five months) in Lwak and from October 2006 to February 2009 (two years and five months) in Kibera.

The PBIDS system has been described in detail [15]. Briefly, it targets infectious disease syndromes, such as diarrheal disease, febrile illness, pneumonia, and jaundice. All residents of all ages in both surveillance sites were eligible for enrollment, as long as they had lived in the region for more than four consecutive months during the study period. Neonates born to a mother enrolled in PBIDS were also offered enrollment.

One unique aspect of the PBIDS system is that it includes both household surveillance and clinic surveillance. In the household surveillance, trained community interviewers visited all enrolled household every two weeks and asked participants about illnesses and deaths that occurred since the last visit using standardized questionnaires. If a participant was absent or unable to answer the questions, a proxy who had the information of the participant’s health was interviewed. Mothers or other primary caretakers were interviewed if children were not able to answer the questions. Community interviewers also conducted focused physical exams on sick participants during the visit. The exam consisted of measuring axillary temperature and respiratory rate, evaluating for signs of respiratory distress in ill children, and observing signs of dehydration. For the clinic surveillance, a centrally located health care facility was identified in each study site, which was located within 5 km or 1 km of all people living in Lwak and Kibera, respectively. All surveillance participants received free medical care at these study clinics for all acute infectious conditions. Detailed information was collected for all sick visits using structured questionnaires and specimens were collected from patients meeting case definitions (see below).

### Specimen collection and diagnostics

At the study clinics, stool specimens were collected from consenting patients of all ages who met at least one of three criteria. The first criterion was uncomplicated diarrhea, which was defined as three or more loose stools within a 24-hour period, without signs or symptoms of dehydration or dysentery. Because there were many patients meeting this criterion, a maximum of six stool specimens were collected from patients with uncomplicated diarrhea per day in each study site: three from patients ≥5 years of age and three from patients <5 years of age. The second criterion was having complicated diarrhea based on the presence of signs and symptoms of dehydration, which was defined as drinking eagerly, being unable to drink or breastfeed, emesis, slow capillary refill (two seconds or more), irritability, sunken eyes, lethargy or unconsciousness. In this group, stool specimens were attempted to be collected from all consenting patients. The third criterion was having dysentery, which was defined as reported or visible blood in one or more stool within 24 hours of clinic visit. We aimed to collect stool specimens from all patients meeting this criterion. If participants were unable to produce stool at the clinic, patients were sent home with stool cups and instructions, and stool specimens were collected from their homes within four hours of clinic visits.

The stool specimens were aliquoted at KEMRI-CDC laboratories in Kenya and stored at −80°C and then an aliquot from each patient was batch shipped to the CDC laboratory in Atlanta, Georgia, on dry ice to test for norovirus, sapovirus, and astrovirus. Viral nucleic acid was extracted from stool suspensions according to the MagMAX-96 Viral RNA Isolation KIT protocol (Ambion, Austin, Texas) on the KingFisher instrument (Thermo Scientific, Vantaa, Finland). Viral nucleic acid was then tested for norovirus genogroup I and II, sapovirus, and astrovirus using TaqMan real-time reverse-transcription polymerase chain reaction (RT-qPCR) [16–20].

In addition to these three enteric viruses, stool specimens were tested for rotavirus using a commercially available enzyme immunoassay kit (Rotaclone, Meridian Premier, Charlotte, North Carolina), as previously described [21], and were cultured for *Salmonella*, *Shigella*, *Campylobacter*, and *Vibrio* species by standard techniques [22].

### Analysis of incidence

Data were analyzed using SAS, version 9.3 (SAS Institute, Cary, North Carolina). Crude incidence was calculated as the number of diarrheal illness episodes with stool specimens testing positive for norovirus, sapovirus, or astrovirus among PBIDS participants per 100,000 participant person-years of observation (PYO). If a patient experienced multiple episodes of diarrhea more than 14 days apart from each other, we considered them as unique episodes and counted them separately in the numerator. PYO was calculated by dividing the sum of person-days for all participants who met the surveillance inclusion criteria by 365.25. A participant who moved away from the surveillance area for longer than four consecutive months was not counted in the denominator for calculation of PYO during or after the leave. If that person returned to the surveillance area, he/she was not counted in denominator or numerator until confirmed to be living in the surveillance area for at least four consecutive months and re-consented to participate in the study.

To assess the seasonality of norovirus, sapovirus, and astrovirus-associated diarrheal illnesses, we calculated annualized crude incidence rates in each month as follows:

$$Annualized\;crude\;incidence\;per\;100,000\;PYO\;in\;each\;month=\frac{Average\;number\;of\;positive\;specimens\;in\;each\;month\;}{PYO}\times 12\times 100,000$$

In order to estimate incidence of the viral diarrhea cases at the outpatient and community levels, we applied three adjustment factors to the crude incidence rates to account for the potentially undetected cases at the study clinics. The first adjustment factor was the proportion of patients whose stool specimens were submitted and tested among all diarrhea cases meeting stool-sampling criteria (P_SS_). This factor accounted for patients who visited the study clinics and met the stool specimen collection criteria, but did not provide a specimen, and patients whose specimens were excluded due to technical issues. The formula below shows how we calculated the adjusted incidence at the study clinic:

$$Study\;clinic\;incidence=\frac{Crude\;incidence}{P_{SS}}$$

The second adjustment factor was the proportion of diarrhea patients who visited the study clinic among diarrhea patients who visited any clinics including non-study clinics (P_SC_). The denominator of this proportion was obtained from the household surveillance, which detected diarrhea patients who visited non-study clinics. The outpatient incidence was calculated as follows:

$$Outpatient\;incidence=\frac{Crude\;incidence}{P_{SS}\times P_{SC}}$$

The third adjustment factor was the proportion of diarrhea patients who visited any clinic among all diarrhea patients identified in the community, including those who did not seek medical care (P_MC_). The household surveillance data enabled us to detect all diarrhea patients in the study sites, and collect information as to whether they accessed any clinic, the determinants of which include a variety of factors. For example, the symptoms were not severe enough to warrant a clinic visit, or simply people made a decision about accessing (or was able to access) a clinic, which may have been unrelated to illness severity. The community incidence was defined as follows:

$$Community\;incidence=\frac{Crude\;incidence}{P_{SS}\times P_{SC}\times P_{MC}}$$

These adjusted incidence rates and their respective 95% credible intervals (CI) were computed using Monte Carlo simulation. We constructed a normal distribution around each of three adjustment factors based on their numerators and denominators, and randomly selected one value from each of the distributions. The crude incidence rates were then divided by these selected values. This process was repeated 100,000 times and the median was reported as the point estimate of adjusted incidence and 2.5^th^ and 97.5^th^ percentile as the 95% CI.

### Ethical approvals

Written informed consent was obtained for data collection at the clinics and households. The protocol, surveillance questionnaires and consent forms were reviewed and approved by the Ethical Review Committee at KEMRI (protocol number 932 and 1899) and the Institutional Review Board of CDC-Atlanta (protocol number 4566).

## Results

### Characteristics of the study population

The patients of this study submitted a total of 336 and 558 stool specimens in Lwak and Kibera, respectively, during the study periods. In Lwak, two stool specimens were excluded from analysis, because these specimens were collected within 14 days since the collection of the initial specimens from the same patients and, thus, considered part of the same episode. In Kibera, a total of 34 stool specimens were excluded from the analysis; 33 were excluded because of missing date of birth, date of specimen collection, or patient ID number, and one was excluded because it was collected within 14 days of another specimen from the same patient. Thus, a total of 334 and 524 stool specimens representing unique episodes of diarrhea were included in the analysis in Lwak and Kibera, respectively. Of these, 18 and 27 patients experienced more than one unique episode of diarrhea during the study periods in Lwak and Kibera, respectively.

Both genders were well represented among the study populations in both sites; 155 (46%) and 257 (49%) episodes of diarrhea were observed among males in Lwak and Kibera, respectively. The surveillance participants in Lwak were older (median age 27.0 years, range 0–85 years) than Kibera (median age 9.6 years, range 0–67 years). Children <5 years of age represented 19% of the study population in Lwak and 40% in Kibera.

### Prevalence of enteric pathogens

In both study sites, norovirus was the most common etiology, detected in 85 (25%) of 334 specimens in Lwak and 159 (30%) of 524 specimens in Kibera (Table 1). Among norovirus positive specimens, genogroup (G) II predominated in both sites, detected in 74 (87%) in Lwak and 140 (88%) in Kibera. Coinfection with GI and GII was found in 6 (7%) norovirus-positive specimens in Lwak and 14 (9%) in Kibera. Overall, the prevalence of sapovirus and astrovirus was low (3–8%) in both study sites. Sapovirus was more prevalent than astrovirus in Kibera, while the opposite was found in Lwak. All viruses were more frequently detected among diarrhea patients aged <5 years compared to those ≥5 years of age in Kibera (p<0.05). The same trend was observed in Lwak, but the difference between the two age groups was not statistically significant.

**Table 1:** Demographic characteristics and detection of enteric viruses among patients with diarrhea, Lwak and Kibera, Kenya.

	Lwak						Kibera					
	Number of unique episodes of diarrhea	NoV GI +	NoV GII +	Any NoV +	Sapovirus +	Astrovirus +	Number of unique episodes of diarrhea	NoV GI +	NoV GII +	Any NoV +	Sapovirus +	Astrovirus +
**Age group, no. (%)**												
<12 monhts	27	1 (4)	9 (33)	10 (37)	2 (7)	5 (19)	43	6 (14)	16 (37)	20 (47)	7 (16)	5 (12)
12–23 months	20	2 (10)	4 (20)	6 (30)	0 (0)	2 (10)	52	5 (10)	15 (29)	16 (31)	4 (8)	5 (10)
24–59 months	17	0 (0)	5 (29)	5 (29)	1 (6)	1 (6)	114	7 (6)	40 (35)	42 (37)	11 (10)	4 (4)
5–9 years	19	2 (11)	4 (21)	5 (26)	0 (0)	4 (21)	57	3 (5)	13 (23)	16 (28)	5 (9)	2 (4)
10–17 years	40	0 (0)	9 (23)	9 (23)	1 (3)	2 (5)	32	0 (0)	11 (34)	11 (34)	0 (0)	0 (0)
18–34 years	98	7 (7)	20 (20)	25 (26)	8 (8)	6 (6)	149	8 (5)	34 (23)	40 (27)	3 (2)	1 (1)
35–49 years	59	5 (8)	14 (24)	16 (27)	1 (2)	4 (7)	60	4 (7)	8 (13)	11 (18)	1 (2)	0 (0)
≥50 years	54	0 (0)	9 (17)	9 (17)	0 (0)	4 (7)	17	0 (0)	3 (18)	3 (18)	0 (0)	1 (6)
**Gender, no. (%)**												
Male	155	5 (3)	42 (27)	46 (30)	7 (5)	10 (6)	257	14 (5)	65 (25)	72 (28)	17 (7)	7 (3)
Female	179	12 (7)	32 (18)	39 (22)	6 (3)	18 (10)	267	19 (7)	75 (28)	87 (33)	14 (5)	11 (4)
**Total, no. (%)**	**334**	**17 (5)**	**74 (22)**	**85 (25)**	**13 (4)**	**28 (8)**	**524**	**33 (6)**	**140 (27)**	**159 (30)**	**31 (6)**	**18 (3)**

NoV GI: norovirus genogroup I; NoV GII: norovirus genogroup II; +: positive.

As shown in Table 2, 311 specimens were positive for at least one of norovirus, sapovirus, or astrovirus. Of these, 28 (9%) and 82 (26%) also tested positive for rotavirus and bacteria, respectively. The proportion of rotavirus coinfection was the highest among patients with complicated diarrhea (9/50, 18%), while coinfection with bacteria occurred most frequently among patients with dysentery (40/84, 48%). Among the 82 specimens in which coinfections of bacteria and at least one of norovirus, sapovirus, or astrovirus were found, *Shigella spp*. (n = 65, 79%) were most frequently identified, followed by *Campylobacter spp*. (n = 9, 11%), and *Salmonella spp*. (n = 7, 9%). Given the potential for asymptomatic (i.e., non-etiologic) detection of norovirus, sapovirus, and astrovirus, we excluded stool specimens from the numerator of crude incidence calculation if they were coinfected with rotavirus and/or bacteria.

**Table 2:** Detection of enteric pathogens stratified by stool specimen collection criteria, Kenya.

	Uncomplicated diarrhea^a^	Complicated diarrhea^b^	Dysentery^c^
	(n = 483)	(n = 101)	(n = 274)
**Single infection, no. (%)**			
NoV	102 (21)	23 (23)	31 (11)
SaV	9 (2)	4 (4)	7 (3)
HAstV	12 (2)	6 (6)	4 (1)
RV	20 (4)	4 (4)	5 (2)
Bacteria	71 (15)	11 (11)	76 (28)
**Coinfection, no. (%)**			
NoV + RV	6 (1)	5 (5)	1 (0)
NoV + Bacteria	25 (5)	2 (2)	24 (9)
NoV + RV + Bacteria	3 (1)	2 (2)	4 (1)
NoV + SaV	4 (1)	0 (0)	0 (0)
NoV + SaV + RV	0 (0)	1 (1)	0 (0)
NoV + SaV + Bacteria	1 (0)	1 (1)	1 (0)
NoV + SaV + HAstV	0 (0)	1 (1)	1 (0)
NoV + HAstV	0 (0)	1 (1)	0 (0)
NoV + HAstV + RV	2 (0)	1 (1)	0 (0)
NoV + HAstV + Bacteria	1 (0)	0 (0)	1 (0)
SaV + HAstV	3 (1)	2 (2)	0 (0)
SaV + RV	2 (0)	0 (0)	0 (0)
SaV + Bacteria	2 (0)	0 (0)	5 (2)
HAstV + RV	1 (0)	0 (0)	0 (0)
HAstV + Bacteria	4 (1)	1 (1)	5 (2)
RV + Bacteria	6 (1)	3 (3)	9 (3)
**No pathogens detected, no. (%)**	209 (43)	33 (33)	100 (36)

NoV: norovirus; SaV: sapovirus; HAstV: astrovirus; RV: rotavirus.

There were 19 specimens that lacked bacteria and/or rotavirus testing, and they are included in the table as bacteria negative and/or rotavirus negative.

Data from two study sites are combined in this table.

^a^ Uncomplicated diarrhea is defined as three or more loose stools within a 24-hour period, without signs or symptoms of dehydration or dysentery.

^b^ Complicated diarrhea is diarrhea with dehydration, which was defined as drinking eagerly, being unable to drink or breastfeed, emesis, slow capillary refill (two seconds or more), irritability, sunken eyes, lethargy or unconsciousness.

^c^ Dysentery is defined as reported or visible blood in one or more stool within 24 hours of clinic visit.

### Clinical profile of norovirus, sapovirus, and astrovirus

The patients with multiple enteric viruses were excluded from the analysis of the clinical profile of norovirus, sapovirus, and astrovirus infections. Uncomplicated diarrhea was most commonly observed among the three types of diarrhea used as the stool collection criteria, followed by dysentery (Table 3). The majority of patients had 3–10 loose stools per day for 1–4 days, regardless of etiology. Vomiting and fever (temperature greater than 37.0°C) were observed in less than half of the patients. No significant difference in clinical profile was observed among the three different viral etiologies.

**Table 3:** Clinical profile of diarrheal patients by etiology, Kenya.

	Norovirus	Sapovirus	Astrovirus
	(n = 156)	(n = 20)	(n = 22)
	No. (%)	No. (%)	No. (%)
**Type of diarrhea**			
Uncomplicated Diarrhea^a^	102 (65)	9 (45)	12 (55)
Complicated Diarrhea^b^	23 (15)	4 (20)	6 (27)
Dysentery^c^	31 (20)	7 (35)	4 (18)
**Diarrhea duration (days)**			
1–4	134 (86)	12 (60)	17 (77)
5	10 (6)	4 (20)	1 (5)
≥6	12 (8)	4 (20)	4 (18)
**Maximum number of stools in 24 hours**^d^			
1–3	5 (9)	2 (33)	0 (0)
3–10	49 (89)	4 (67)	5 (100)
≥11	1 (2)	0 (0)	0 (0)
**Vomit**			
Yes	41 (35)	5 (42)	7 (35)
No	77 (65)	7 (58)	13 (65)
**Maximum temperature (°C)**			
≤37.0	117 (76)	15 (75)	13 (59)
37.1–38.4	26 (17)	3 (15)	6 (27)
38.5–38.9	4 (3)	0 (0)	1 (5)
≥39.0	6 (4)	2 (10)	2 (9)
**Dehydration**			
Yes	23 (15)	4 (20)	6 (27)
No	133 (85)	16 (80)	16 (73)

Patients with multiple enteric viruses were excluded from the table.

Data from two study sites are combined in this table.

^a^ Uncomplicated diarrhea is defined as three or more loose stools within a 24-hour period, without signs or symptoms of dehydration or dysentery.

^b^ Complicated diarrhea is diarrhea with dehydration, which was defined as drinking eagerly, being unable to drink or breastfeed, emesis, slow capillary refill (two seconds or more), irritability, sunken eyes, lethargy or unconsciousness.

^c^ Dysentery is defined as reported or visible blood in one or more stool within 24 hours of clinic visit.

^d^ The data on maximum number of stool specimens was not available for Lwak.

### Crude and adjusted incidence of diarrheal illness

**Fig 2:**
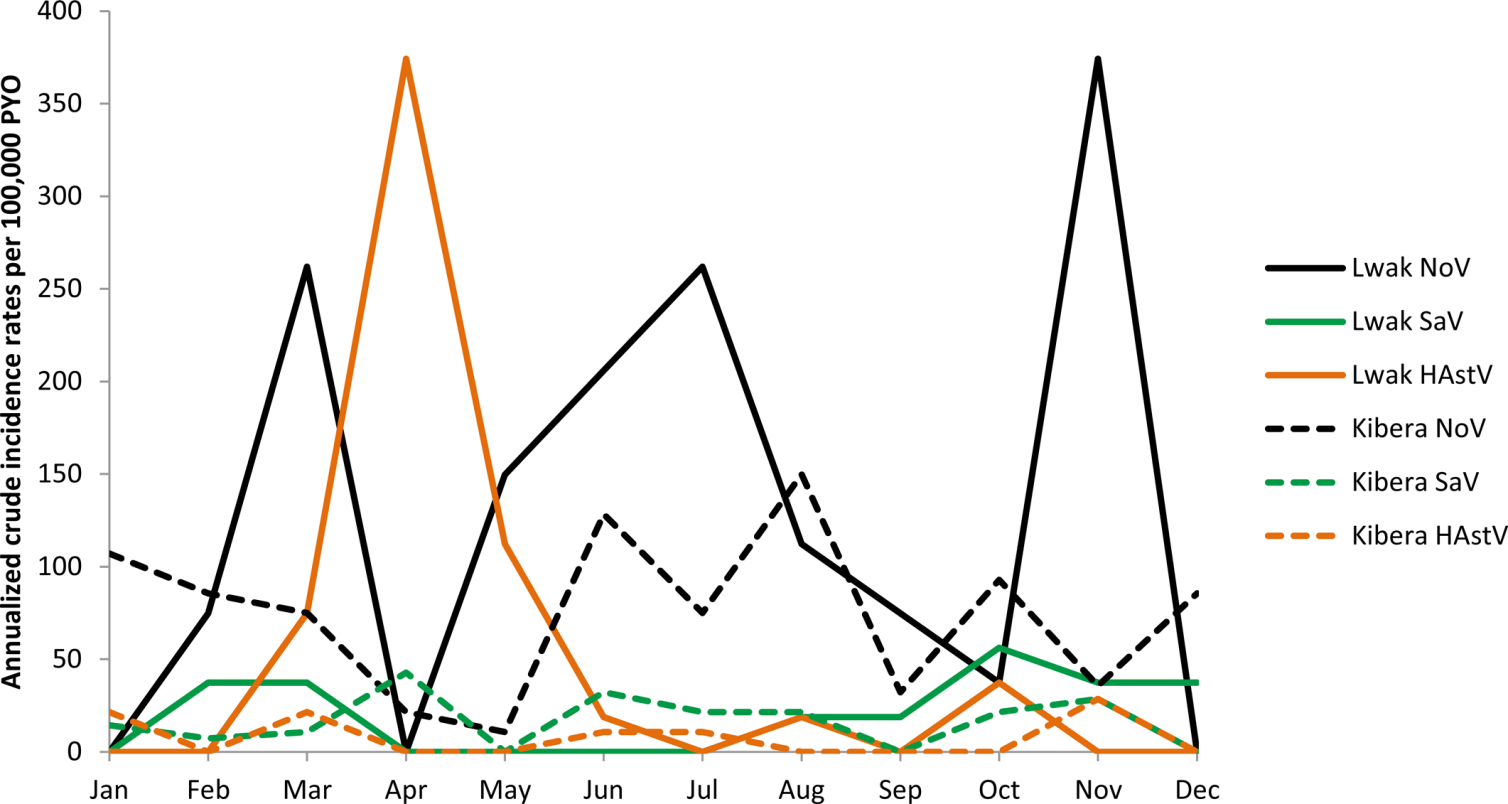
Annualized crude incidence rates of enteric viruses per 100,000 PYO by month, Lwak and Kibera, Kenya. NoV: norovirus; SaV: sapovirus; HAstV: astrovirus; PYO: person-years of observation. Study period was from June 2007 to October 2008 in Lwak and from October 2006 to February 2009 in Kibera. Data in the same months were combined together and annualized crude incidence rates per 100,000 PYO in each month were calculated as follows:
$$\frac{Average\;number\;of\;positive\;specimens\;in\;each\;month}{PYO}\times 12\times 100,000.$$

The overall crude incidence rates of norovirus, sapovirus, and astrovirus-associated diarrheal illnesses were 187, 28, and 59 per 100,000 PYO in Lwak (Table 4) and 184, 39, and 20 per 100,000 PYO in Kibera (Table 5). The crude incidence rates of all viruses were the highest among children <12 months of age in both sites. As shown in Fig 2, there was no clear seasonality of crude incidence rates of diarrheal illness associated with these enteric viruses. The annualized crude incidence of norovirus and astrovirus exhibited a large peak in November and April, respectively, in Lwak, but a similar trend by month was not observed in Kibera. The annualized crude incidence of sapovirus was low throughout the year in both sites.

**Table 4:** Crude and adjusted incidence rates for norovirus, sapovirus, and astrovirus-associated diarrheal illness, Lwak, Kenya.

					Adjustment 1		Adjustment 2			Adjustment 3		
		Number of AGE cases positive for each virus	PYO	Crude incidence	% cases with stool specimen (P_SS_)	Study clinic incidence per 100,000 PYO	% cases visiting study clinics (P_SC_)	Outpatient incidence per 100,000 PYO	(95% CI)	% cases visiting any clinic (P_MC_)	Community incidence per 100,000 PYO	(95% CI)
**Age group**												
<12 months	NoV	5	1049	477	6.2	7700	54.3	14178	(10507 to 21589)	40.7	34900	(25669 to 53294)
	SaV	2	1049	191	6.2	3080	54.3	5671	(4203 to 8635)	40.7	13960	(10268 to 21318)
	HAstV	3	1049	286	6.2	4620	54.3	8507	(6304 to 12953)	40.7	20940	(15402 to 31976)
12–23 months	NoV	4	953	420	7.0	5955	50.8	11736	(8486 to 18663)	33.2	35308	(25477 to 56276)
	SaV	0	953	0	7.0	0	50.8	0		33.2	0	
	HAstV	2	953	210	7.0	2978	50.8	5868	(4243 to 9331)	33.2	17654	(12738 to 28138)
24–59 months	NoV	3	2836	106	5.8	1822	53.1	3438	(2412 to 5892)	27.7	12423	(8669 to 21370)
	SaV	1	2836	35	5.8	607	53.1	1146	(804 to 1964)	27.7	4141	(2890 to 7123)
	HAstV	1	2836	35	5.8	607	53.1	1146	(804 to 1964)	27.7	4141	(2890 to 7123)
5–9 years	NoV	5	5357	93	11.6	804	50.5	1596	(1131 to 2621)	24.4	6554	(4591 to 10860)
	SaV	0	5357	0	11.6	0	50.5	0		24.4	0	
	HAstV	4	5357	75	11.6	643	50.5	1277	(905 to 2097)	24.4	5243	(3673 to 8688)
10–17 years	NoV	8	6662	120	13.5	887	53.3	1666	(1281 to 2341)	23.9	6992	(5287 to 9924)
	SaV	1	6662	15	13.5	111	53.3	208	(160 to 293)	23.9	874	(661 to 1241)
	HAstV	1	6662	15	13.5	111	53.3	208	(160 to 293)	23.9	874	(661 to 1241)
18–34 years	NoV	16	7229	221	24.8	892	53.1	1682	(1419 to 2053)	28.9	5832	(4838 to 7166)
	SaV	4	7229	55	24.8	223	53.1	421	(355 to 513)	28.9	1458	(1210 to 1792)
	HAstV	4	7229	55	24.8	223	53.1	421	(355 to 513)	28.9	1458	(1210 to 1792)
35–49 years	NoV	11	3347	329	23.5	1400	53.5	2622	(2102 to 3423)	26.3	9963	(7885 to 13172)
	SaV	1	3347	30	23.5	127	53.5	238	(191 to 311)	26.3	906	(717 to 1197)
	HAstV	3	3347	90	23.5	382	53.5	715	(573 to 933)	26.3	2717	(2150 to 3592)
≥50 years	NoV	8	4636	173	21.7	793	52.2	1523	(1227 to 1981)	17.6	8661	(6915 to 11344)
	SaV	0	4636	0	21.7	0	52.2	0		17.6	0	
	HAstV	1	4636	22	21.7	99	52.2	190	(153 to 248)	17.6	1083	(864 to 1418)
<5 years	NoV	12	4838	248	6.3	3920	52.8	7430	(6094 to 9471)	33.4	22225	(18184 to 28432)
	SaV	3	4838	62	6.3	980	52.8	1858	(1524 to 2368)	33.4	5556	(4546 to 7108)
	HAstV	6	4838	124	6.3	1960	52.8	3715	(3047 to 4736)	33.4	11113	(9092 to 14216)
≥5 years	NoV	48	27231	176	19.7	893	52.6	1697	(1527 to 1905)	23.1	7336	(6566 to 8264)
	SaV	6	27231	22	19.7	112	52.6	212	(191 to 238)	23.1	917	(821 to 1033)
	HAstV	13	27231	48	19.7	242	52.6	460	(414 to 516)	23.1	1987	(1778 to 2238)
**Sex**												
Male	NoV	31	15177	204	12.7	1603	52.1	3073	(2674 to 3598)	28.3	10856	(9394 to 12754)
	SaV	4	15177	26	12.7	207	52.1	397	(345 to 464)	28.3	1401	(1212 to 1646)
	HAstV	6	15177	40	12.7	310	52.1	595	(517 to 696)	28.3	2101	(1818 to 2468)
Female	NoV	29	16892	172	14.3	1199	53.2	2256	(1988 to 2601)	26.2	8594	(7545 to 9946)
	SaV	5	16892	30	14.3	207	53.2	389	(343 to 448)	26.2	1482	(1301 to 1715)
	HAstV	13	16892	77	14.3	538	53.2	1011	(891 to 1166)	26.2	3852	(3382 to 4459)
**Total**	NoV	60	32069	187	13.6	1380	52.7	2619	(2380 to 2907)	27.2	9635	(8733 to 10717)
	SaV	9	32069	28	13.6	207	52.7	393	(357 to 436)	27.2	1445	(1310 to 1608)
	HAstV	19	32069	59	13.6	437	52.7	829	(754 to 920)	27.2	3051	(2766 to 3394)

NoV: norovirus; SaV: sapovirus; HAstV: astrovirus; PYO: person-years of observation; AGE: acute gastroenteritis; 95% CI: 95% credible interval.

**Table 5:** Crude and adjusted incidence rates for norovirus, sapovirus, and astrovirus-associated diarrheal illness, Kibera, Kenya.

					Adjustment 1		Adjustment 2			Adjustment 3		
		Number of AGE cases positive for each virus	PYO	Crude incidence	% cases with stool specimen (P_SS_)	Study clinic incidence per 100,000 PYO	% cases visiting study clinics (P_SC_)	Outpatient incidence per 100,000 PYO	(95% CI)	% cases visiting any clinic (P_MC_)	Community incidence per 100,000 PYO	(95% CI)
**Age group**												
<12 months	NoV	16	1543	1037	5.2	19785	76.8	25768	(20930 to 33448)	57.2	45072	(36395 to 58670)
	SaV	5	1543	324	5.2	6183	76.8	8053	(6541 to 10453)	57.2	14085	(11373 to 18334)
	HAstV	5	1543	324	5.2	6183	76.8	8053	(6541 to 10453)	57.2	14085	(11373 to 18334)
12–23 months	NoV	11	2237	492	6.1	8057	82.9	9718	(8317 to 11650)	50.0	19452	(16577 to 23420)
	SaV	3	2237	134	6.1	2197	82.9	2650	(2268 to 3177)	50.0	5305	(4521 to 6387)
	HAstV	3	2237	134	6.1	2197	82.9	2650	(2268 to 3177)	50.0	5305	(4521 to 6387)
24–59 months	NoV	29	6406	453	10.8	4180	83.0	5037	(4515 to 5687)	43.5	11590	(10311 to 13182)
	SaV	6	6406	94	10.8	865	83.0	1042	(934 to 1177)	43.5	2398	(2133 to 2727)
	HAstV	1	6406	16	10.8	144	83.0	174	(156 to 196)	43.5	400	(356 to 455)
5–9 years	NoV	12	8485	141	17.6	801	84.7	946	(809 to 1136)	44.1	2145	(1804 to 2615)
	SaV	5	8485	59	17.6	334	84.7	394	(337 to 473)	44.1	894	(752 to 1090)
	HAstV	1	8485	12	17.6	67	84.7	79	(67 to 95)	44.1	179	(150 to 218)
10–17 years	NoV	8	8582	93	22.8	410	84.7	484	(400 to 609)	41.0	1186	(946 to 1533)
	SaV	0	8582	0	22.8	0	84.7	0	(0 to 0)	41.0	0	(0 to 0)
	HAstV	0	8582	0	22.8	0	84.7	0	(0 to 0)	41.0	0	(0 to 0)
18–34 years	NoV	19	20698	92	20.5	447	73.0	612	(541 to 704)	42.3	1451	(1254 to 1693)
	SaV	2	20698	10	20.5	47	73.0	64	(57 to 74)	42.3	153	(132 to 178)
	HAstV	1	20698	5	20.5	24	73.0	32	(28 to 37)	42.3	76	(66 to 89)
35–49 years	NoV	5	6660	75	25.5	294	67.8	435	(366 to 527)	39.9	1092	(893 to 1361)
	SaV	1	6660	15	25.5	59	67.8	87	(73 to 105)	39.9	218	(179 to 272)
	HAstV	0	6660	0	25.5	0	67.8	0	(0 to 0)	39.9	0	(0 to 0)
≥50 years	NoV	3	1458	206	18.5	1112	79.0	1416	(998 to 2310)	42.5	3347	(2247 to 5630)
	SaV	0	1458	0	18.5	0	79.0	0	(0 to 0)	42.5	0	(0 to 0)
	HAstV	0	1458	0	18.5	0	79.0	0	(0 to 0)	42.5	0	(0 to 0)
<5 years	NoV	56	10187	550	7.9	6981	81.1	8604	(7908 to 9427)	49.1	17511	(16042 to 19250)
	SaV	14	10187	137	7.9	1745	81.1	2151	(1977 to 2357)	49.1	4378	(4011 to 4812)
	HAstV	9	10187	88	7.9	1122	81.1	1383	(1271 to 1515)	49.1	2814	(2578 to 3094)
≥5 years	NoV	47	45883	102	20.9	491	77.3	636	(588 to 690)	42.2	1506	(1379 to 1651)
	SaV	8	45883	17	20.9	84	77.3	108	(100 to 117)	42.2	256	(235 to 281)
	HAstV	2	45883	4	20.9	21	77.3	27	(25 to 29)	42.2	64	(59 to 70)
**Sex**												
Male	NoV	51	28442	179	11.9	1508	80.0	1885	(1738 to 2056)	47.4	3979	(3649 to 4360)
	SaV	12	28442	42	11.9	355	80.0	443	(409 to 484)	47.4	936	(859 to 1026)
	HAstV	3	28442	11	11.9	89	80.0	111	(102 to 121)	47.4	234	(215 to 256)
Female	NoV	52	27629	188	11.8	1598	80.3	1991	(1836 to 2172)	46.7	4260	(3910 to 4669)
	SaV	10	27629	36	11.8	307	80.3	383	(353 to 418)	46.7	819	(752 to 898)
	HAstV	8	27629	29	11.8	246	80.3	306	(283 to 334)	46.7	655	(602 to 718)
**Total**	NoV	103	56070	184	11.8	1552	80.1	1937	(1828 to 2059)	47.1	4116	(3871 to 4389)
	SaV	22	56070	39	11.8	331	80.1	414	(390 to 440)	47.1	879	(827 to 938)
	HAstV	11	56070	20	11.8	166	80.1	207	(195 to 220)	47.1	440	(413 to 469)

NoV: norovirus; SaV: sapovirus; HAstV: astrovirus; PYO: person-years of observation; AGE: acute gastroenteritis; 95% CI: 95% credible interval.

**Fig 3:**
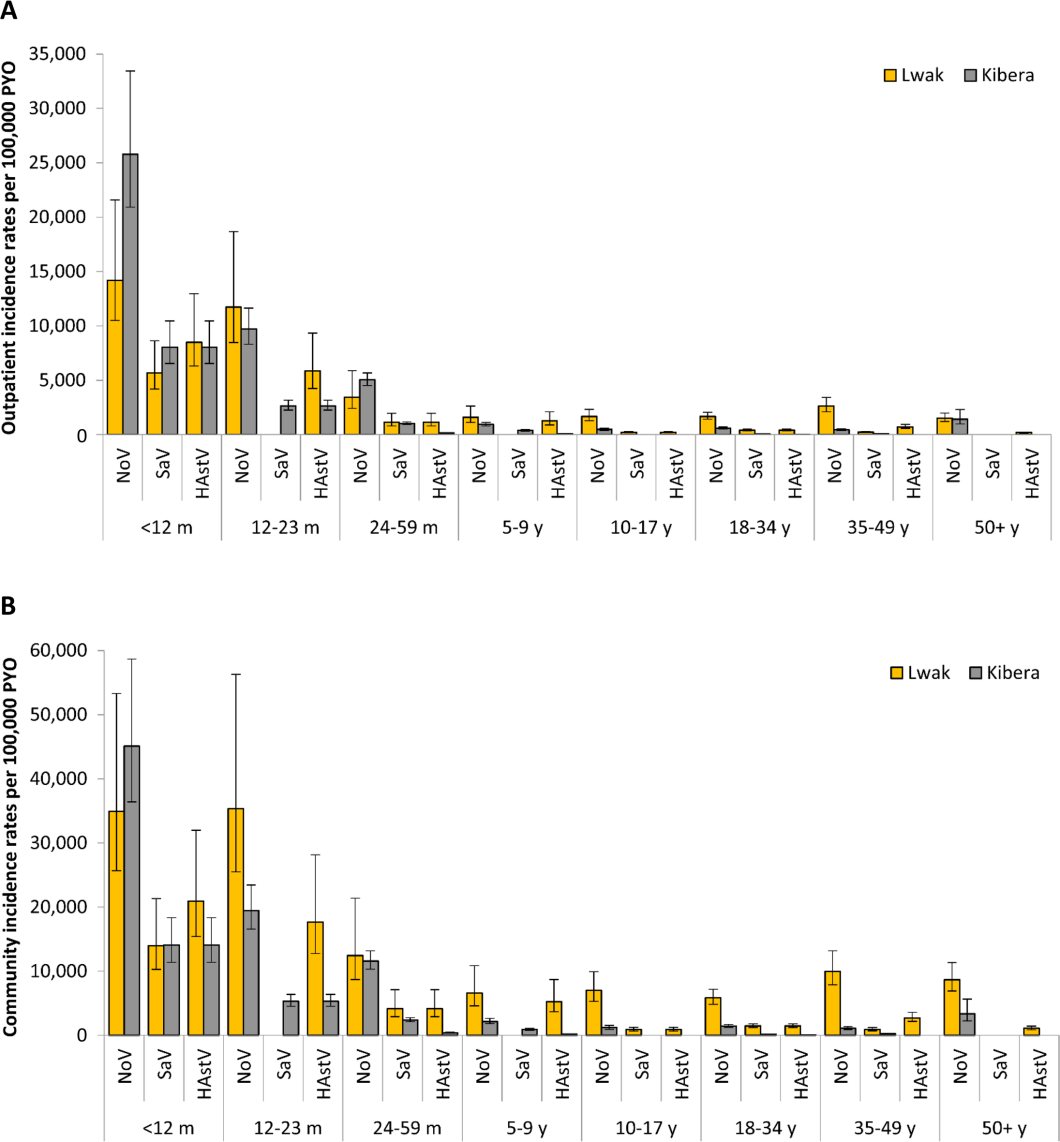
**Adjusted outpatient (A) and community (B) incidence rates of enteric viruses per 100,000 PYO by age group, Lwak and Kibera, Kenya.** NoV: norovirus, SaV: sapovirus, HAstV: astrovirus; PYO: person-years of observation. The outpatient incidence was calculated by dividing the crude incidence by the proportion of patients who submitted stool specimen among all diarrhea patients meeting stool-sampling criteria (P_SS_) and the proportion of diarrhea patients who visited the study clinic among diarrhea patients who visited any clinics including non-study clinics (P_SC_). The community incidence was calculated by dividing the outpatient incidence by the proportion of diarrhea patients who visited any clinics among all diarrhea patients identified in the community, including those who did not seek medical care (P_MC_).

The proportion of diarrhea patients with a stool specimen (P_SS_) was low in both sites (14% in Lwak and 12% in Kibera), and appeared to be even lower among younger children (Tables 4 and 5). About half (53%) of the patients who sought medical attention in Lwak and 80% of them in Kibera presented at the study clinics (P_SC_). After adjusting the crude incidence rates by P_SS_ and P_SC_, the overall outpatient incidence rates of norovirus, sapovirus, and astrovirus-associated diarrheal illnesses became 2,619, 393, and 829 per 100,000 PYO in Lwak and 1,937, 414, and 207 per 100,000 PYO in Kibera. In both sites, norovirus was the most prevalent among all age groups. The highest outpatient incidence rates for all viruses detected were observed among children <12 months of age (Fig 3A). The outpatient incidence associated with detection of norovirus decreased by 17% in Lwak and 62% in Kibera in children 12–23 months of age compared to children <12 months of age.

The overall healthcare seeking rate for acute diarrheal illness (P_MC_) was lower in Lwak (27%) versus Kibera (47%) and decreased as age increased in both sites (Tables 4 and 5). By adjusting the outpatient incidence rates by P_MC_, the overall community incidence rates per 100,000 PYO of all viruses were found to be higher in Lwak (norovirus: 9,635; sapovirus: 1,445; astrovirus: 3,051) than in Kibera (norovirus: 4,116; sapovirus: 879; astrovirus: 440). Overall, the community incidence rates of all viruses were the highest among children aged <24 months and decreased as age increased (Fig 3B). In Kibera, the community incidence of all viruses were low among people aged ≥5 years, but an increase in norovirus was observed among people aged ≥50 years.

## Discussion

To our knowledge, this is the first study to estimate the outpatient and community incidence rates of norovirus, sapovirus, and astrovirus-associated diarrheal illnesses across the whole age spectrum in Africa. Our analysis demonstrated a substantial disease burden imposed by these viruses in both rural and urban Kenya. Norovirus had the highest incidence rates in both sites. In addition, sapovirus and astrovirus, for which epidemiological data remain limited globally, were also found to be of considerable disease burden in Kenya. The combination of household surveillance and clinic surveillance enabled us to identify patients with diarrhea in the study sites comprehensively, and provided robust estimates of incidence at the outpatient and community levels by adjusting for the proportion of patients that were missed at the study clinics. These adjustments revealed the total community burden of disease associated with each virus, which could not have been observed from the crude incidence rates alone. For example, the overall crude incidence of norovirus was about same in Kibera than in Lwak, but the overall outpatient and community incidence rates were calculated to be about 1.4 and 2.3-fold higher in Lwak than in Kibera, respectively. These findings demonstrated the importance of assessing healthcare seeking behaviors and access to care issues in the context of surveillance studies for disease burden estimates. We did not detect an apparent or consistent trend in seasonality at either study site. Both study sites have two rainy seasons (from March to May and from October to November) [15], but no viruses at either of the sites peaked during both rainy seasons.

According to the 2009 census, there were 26.1 million and 12.5 million people of all ages living in rural and urban Kenya, respectively [23]. By applying the adjusted incidence rates in Lwak to the population in rural Kenya and those in Kibera to the population in urban Kenya, we estimate that each year, about 2.8–3.3 million, 0.45–0.54 million, and 0.77–0.95 million people across all ages in Kenya become ill with diarrheal illness in the presence of norovirus, sapovirus, and astrovirus, respectively. Of these, approximately 0.85–1.0 million, 0.14–0.17 million, and 0.22–0.27 million people of all ages with norovirus, sapovirus, and astrovirus-associated diarrheal illnesses seek outpatient care each year in Kenya, respectively. These extrapolations are based on the assumption that the incidence estimated in the single rural and urban study sites reflect the incidence in all rural and urban areas of Kenya, respectively. This assumption may not be appropriate especially for urban areas where the population size is not stable because of frequent movement in and out of the area. Due to this assumption and other limitations discussed later, these extrapolations may not yield precise national estimates. Nonetheless, the magnitude of these estimates suggests the significant burden of these enteric viruses on the people and healthcare system in Kenya and the importance of targeted public health interventions for these enteric viruses. Compared to the outpatient incidence of rotavirus estimated in the same sites among children aged <5 years from 2007 to 2010 (before introduction of rotavirus vaccine into the national immunization program in 2014) [14], the outpatient incidence of norovirus was estimated to be 10% higher in Lwak and 46% higher in Kibera among the same age group. Among the total population across all age groups, the outpatient incidence of norovirus was about 2-fold higher than that of rotavirus in both sites. Although rotavirus was detected by enzyme immunoassay in the previous study, which is less sensitive than RT-PCR, these findings suggest that norovirus is comparably or even more prevalent than rotavirus among diarrhea patients in the outpatient setting. As diarrheal disease caused by rotavirus decreases with the introduction of rotavirus vaccines [24, 25], the relative burden of norovirus is expected to increase in the future.

Comparison of our study findings with similar studies conducted in developed countries reveals consistent trends and key differences. For example, the estimated community incidence rates of norovirus disease per 100,000 population across all age groups was 6,500–7,000 in the U.S. [26–28], 3,800 in the Netherlands [29], and 4,500–4,700 in the United Kingdom [30, 31]. Although these estimates were computed by various methods, rates of norovirus-associated diseases appear to be 1.2–2.1-fold higher in Kenya compared to those in developed countries. With regard to adjustment factors for healthcare utilization, the rate of stool specimen submission was low (5–11%) among younger children and was the highest (21–26%) among patients aged 18–49 years in both sites in Kenya (Table 3). This is contrary to a previous study’s observation in the U.S., where young children and the elderly were more likely to submit stool specimens and only less than 1% of patients aged 16–25 years provided specimens [28]. This observation highlights the importance of obtaining the site specific adjustment factors in order to estimate an outpatient and community incidence of diseases.

An important limitation of this study is the lack of non-diarrheal or healthy control specimens. As such, we were not able to adjust the attributable fraction to account for asymptomatic viral detection, as has been done previously [14]. However, we conservatively limited our incidence estimates for norovirus, sapovirus, and astrovirus to only include diarrhea patients that were not positive for another known pathogen (i.e., enteric bacteria or rotavirus), providing some reassurance that these were etiologic detections. Other studies have detected these viruses more frequently in specimens collected from cases compared to those from controls, suggesting that these three viruses are indeed etiologic causes of diarrheal illnesses [32–34]. It should be also noted that specimens were not tested for other causes of diarrhea, such as parasites and adenovirus. Although norovirus was detected as a single pathogen in 11% of dysentery cases, these cases may have been caused by other pathogens that were not tested in our study or may represent false negatives of the bacteria testing. There is also a potential that the clinic surveillance missed patients with mild diarrhea, because we did not collect stool specimens from all patients who experienced uncomplicated diarrhea. As norovirus is known to more often cause less severe diarrhea, some cases with norovirus-associated uncomplicated diarrhea were likely missed at the study clinics, possibly causing an underestimation of our incidence rates. Another potential limitation is selection bias among patients that submitted stool specimens and sought medical care at the study clinics, which would limit the representativeness of the data on viral detection from stools. In addition, because our study periods were not a set on full calendar years, there is potential for seasonal bias; however, our study was performed for at least one full calendar year in both sites and our analysis showed there was no clear seasonality in either site.

In conclusion, our study is the first to provide population-based incidence estimates of diarrheal illness associated with norovirus, sapovirus, and astrovirus across the age spectrum in Kenya, and one of the few studies providing such estimates worldwide. The substantial disease burden of norovirus, which is comparable to that of rotavirus, underscores the potential value of targeted interventions, such as vaccines, to reduce the health burden of norovirus. Data from our study can inform cost-effectiveness of potential vaccines against norovirus and help define a public health strategy for controlling these enteric viruses in developing countries.
